# The impact of the national volume-based procurement policy on the use of policy-related drugs in Nanjing: an interrupted time-series analysis

**DOI:** 10.1186/s12939-023-02006-1

**Published:** 2023-09-28

**Authors:** Xiao Wang, Xuan He, Pei Zhang, Mengdie Zhang, Rui Ma, Rouli Dai, Xin Li

**Affiliations:** 1 School of Health Policy and Management, Nanjing Medical University, No.101 Longmian Avenue, Jiangning District, Nanjing, Jiangsu P.R. China 211166; 2 Department of Science and Technology, Wuxi No.2 People’s Hospital, Wuxi, China; 3 Wuxi Xinwu District Center for Health Promotion and Education, Wuxi, China; 4 School of Pharmacy, Nanjing Medical University, Nanjing, China; 5 National Institute of Drug Clinical Trials, the Fourth Affiliated Hospital of Nanjing Medical University, Nanjing, China; 6 Center for Global Health, School of Public Health, Nanjing Medical University, Nanjing, China

**Keywords:** National volume-based procurement policy, Interrupted time series, Drug use, Alternative drugs, Centralized procurement

## Abstract

**Background:**

In September 2019, the “4 + 7” centralized procurement pilot program was expanded nationwide aiming at reducing drug prices by means of volume-based procurement and using accredited generic drugs for branded drug substitutes. Given the current uncertain effect of the policy outside pilot areas, this study was conducted to evaluate the impact of the National Volume-based Procurement policy on the use of policy-related drugs after expansion.

**Method:**

A single-group interrupted time series was applied using drug purchase data, covering 25 months from December 2018 to December 2020. Drugs related to the centralized procurement policy were selected as samples, including 25 first-batch policy-related drugs and 56 alternative drugs. Centralized procured drugs can be divided into bid-winning and non-winning products, where non-winning products were sorted into generic and branded drugs, and alternative products were classified according to different degrees of substitution. Purchase volume, expenditures, and daily costs were measured.

**Results:**

After the implementation of the policy, a significant increase was associated with the volume of bid-winning drugs (*p* < 0.001) and the volume of generic and branded drugs decreased immediately. The DDDc of drugs under the same generic name significantly reduced (an instantaneous drop of bid-winning drugs by approximately 25%, 7.62 CNY for generics and 3.07 CNY for branded drugs), saving 48.2 million CNY of drug expenditures. The policy has a significant effect on the drug for the treatment of cardiovascular diseases and exerted little influence on the drug for the treatment of nervous diseases, and the substitution of generics for antitumor-branded drugs was not obvious. In addition, the procurement volume of alternative drugs appeared to be a “carry-over”.

**Conclusions:**

These findings indicated that the policy demonstrated positive effects in terms of price reductions and cost savings and accelerated the substitution of generics against branded drugs. The “patent cliff” for branded drugs has gradually emerged. Besides, a short-term “spillover effect” of the volume of alternative drugs was observed, requiring special attention and vigilance.

**Supplementary Information:**

The online version contains supplementary material available at 10.1186/s12939-023-02006-1.

## Introduction

As the largest developing country worldwide, China is facing unprecedented challenges in improving the healthcare system, such as population aging, rising healthcare spending, and health inequities between urban and rural areas [1]. To provide universal health coverage (UHC) to all citizens, China has launched a nationwide systematic healthcare reform since 2009 [2]. Until 2022, the health reform has expanded the basic healthcare insurance coverage to about 96% of the population, which benefits 1.36 billion citizens in China [1]. China has also built an integrated healthcare system to improve the accessibility of medical resources. However, China’s healthcare budget is gradually increasing in recent years. During the period from 2000 to 2010, the ratio of health expenditures to GDP was only 4.3% to 5.2%, yet it increased to 6.5% in 2021 [1]. In recent years, the government continuously expanded health expenditures, which quadrupled in 2017 compared with 2008 [2]. The total national health expenditure has reached 1184.9 billion dollars in 2021 [3]. In particular, pharmaceuticals take up a large proportion, accounting for about 31% of the nation’s total health expenditures in 2020 [4]. The high drug prices, which increase the heavy medication burden of patients, have been a prominent problem in the construction of China's medical system, possibly due to China's drug procurement mechanism. Therefore, more and more measures have been conducted to curb the skyrocketing drug costs. The Centralised Procurement of Medicine Policy (CPMP) was established in the Chinese healthcare system to strengthen the bargaining power of strategic purchaser and reduce the purchase prices of drugs [5]. Prior to 2018, China conducted spontaneous or joint drug procurement on a provincial or municipal basis. However, these mechanisms appear to be less effective than expected, which may be attributed to the widespread “separation of bidding and procurement and the decoupling of volume and price”. On the other hand, in order to ensure equitable access to safe, effective, and affordable drugs, China’s healthcare authorities tried to use the lowest-priced generic drugs to improve the health of residents.

After years of reforms that tried to lower drug prices, the National Healthcare Security Administration (NHSA) launched the national volume-based procurement policy (NVBP) in December 2018 [6]. As an important part of promoting the reform of medicine, medical insurance, and public medical institutions, the NVBP was first piloted in the “4 + 7” cities, including four municipalities (Beijing, Shanghai, Tianjin, and Chongqing) and seven cities in other provinces (Xi’an, Dalian, Guangzhou, Chengdu, Shenzhen, and Xiamen). Under the “4 + 7” program, the government awarded a contract to the lowest bidder, who will be guaranteed a sale volume of 60–70% of the total market for a year. The aims of policy-makers are to ensure the priority use of bid-winning drugs and encourage pharmaceutical enterprises to reduce drug prices. Essentially, as the agent of a group of buyers negotiates, the government can help the buyers to form one purchasing unit in the transparent bidding and procurement platform. It is beneficial for the buyers to get lower purchasing prices by establishing collective bargaining power [5]. In the context of NVBP policy, it is unnecessary for pharmaceutical enterprises to provide kickbacks to doctors to promote the sales volume of drugs. However, as an old Chinese saying, “Rob Peter to pay Paul”. Could the priority use of bid-winning drugs lead to a rapid increase in the volume of their alternative drugs? In other words, in order to get the kickbacks from other drugs, the doctors could choose to prescribe some unnecessary alternative drugs for patients. Unfortunately, there is still no clear answer to the above question.

Subsequently, the policy was extended to the whole country, with more than 20 provinces and regions forming alliances to join the centralized procurement. The highlight of this policy is that it is the first nationwide attempt at a “volume-based drug procurement” policy in China, which specifies the varieties and quantities of drugs to be procured and determines approximately 60–70% of the dosage. It aims to achieve the objectives of replacing generic drugs with originators, eliminating pharmaceutical rebates, guiding drug prices to a reasonable level, and optimizing the structure of drug use.

Centralized drug procurement is a systematic project, involving the whole process of production, trading, circulation, and, use of drugs. In order to ensure the policy is truly implemented, the government has made an unprecedented high political commitment: (1) Generic drugs that pass the Generic Consistency Evaluation (GCE) are a prerequisite to be eligible for inclusion on the procurement list, which guarantees the quality of the bid-winning drugs to a certain extent [5, 7]. (2) The bid-winning drugs were listed in the key regulatory varieties and strengthened the supervision of production and distribution of bid-winning drug enterprises to ensure that doctors dare to use the bid-winning drugs and the supply of drugs was adequate. (3) To ensure that the bid-winning drugs are given priority, requirements for clinical prescriptions of medical institutions and doctors have been put forward, and incorporate the procurement and use of bid-winning drugs by medical institutions into the performance appraisal system for public hospitals [8].

Previous studies revealed the implications from different perspectives after the implementation of centralized drug procurement: for instance, the policy promoted the substitution use of generic drugs against original drugs and drug consumption has gradually concentrated on bid-winning drugs, generic drugs, and quality-guaranteed drugs [9, 10]. In China, the cost of drugs is shared between individuals and the government on a proportional basis according to the National Reimbursement Drug List (NRDL) to which the drugs belong. The NRDL is divided into three categories: A, B, and C, of which class A drugs are uniformly formulated by the State, necessary for clinical treatment, widely used and with good curative effect, and are fully included in the scope of reimbursement; Class B drugs require individuals to pay a certain percentage of costs after reimbursement, which means that health insurance covers part of the costs of Class B drugs; Class C drugs are self-paid drugs and need to be fully paid by individuals [11]. By analyzing the drug prices of 25 bid-winning drugs and their alternative drugs in 11 pilot cities, Long, H. revealed that the policy facilitated drug price cuts and alleviated healthcare pressure, and a short-term “spillover” effect of synergistic price reduction was also observed [12]. Relevant studies also reported the prominently increased use of bid-winning drugs after policy intervention [8, 13].

In China, drug utilization condition varies between different geographical regions, healthcare facilities, and drug therapeutic categories [14]. In this program, the 25 drugs in the procurement list can be divided into 8 categories by generic name, the impact of the policy might cover patients with a variety of diseases. In addition, we should pay more attention to alternative drugs. Monitoring the utilization of alternative drugs, on the one hand, can effectively mitigate the risk of a shortage of drugs in the process of centralized drug procurement. On the other hand, it can also reflect the actual implementation of the NVBP. However, most studies at this stage were focused on pilot cities and the relevant evidence mainly came from therapeutic drugs for certain types of diseases or descriptive statistical analysis of the macroscopic results. In light of this, we conducted a systematic analysis to demonstrate changes in drug utilization of bid-winning drugs in different therapeutic categories and their alternative drugs at different degrees. Therefore, our study's contributions and objectives were: (1) To quantitatively evaluate the impact of NVBP on the volume, daily cost, and expenditures of policy-related drugs. (2) To assess whether the policy has improved the accessibility and affordability of patient medication. (3) To provide suggestions for further improving the NVBP and references for international drug policy research.

## Methods

### Data source

In this study, the research site is one of the “4 + 7” pilot expansion cities –Nanjing, the capital of Jiangsu Province. Nanjing is a key central city in Eastern China and it forms part of the important gateway cities in the Yangtze River Delta. It is located at the strategic junction with the eastern coastal economic belt and is the central hub of the central and western regions. We conducted a retrospective examination of monthly data on drug procurement reported from 60 hospitals in Nanjing to quantitatively evaluate the volume, expenditures, and daily cost of policy-related drugs and alternative drugs. The data used in this study came from the Jiangsu Medicine Information Institute, covering the drug procurement order data of 60 hospitals in Nanjing (24 tertiary hospitals,6 secondary hospitals, and 30 primary hospitals), which exhibited great authenticity, and representativeness.

### Drug classification

**Fig. 1:**
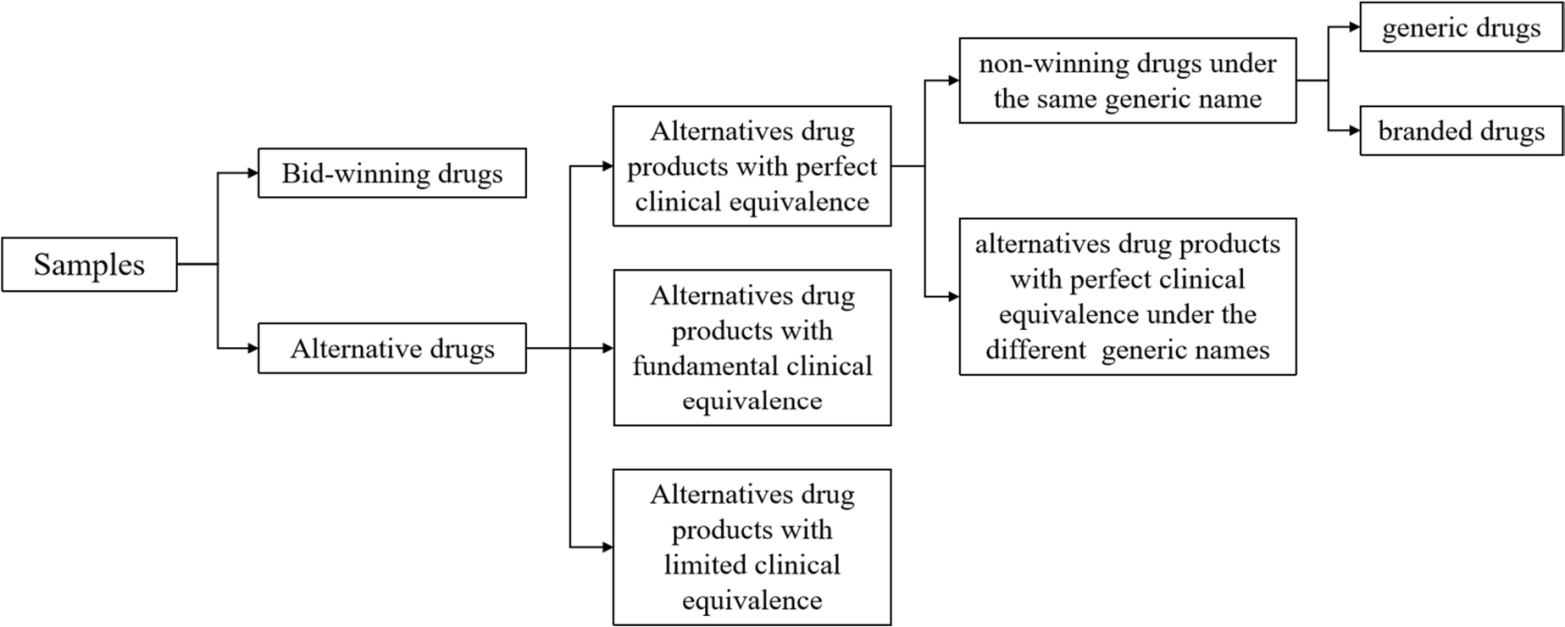
The classification of surveyed drugs and the relationship between bid-winning drugs and alternative drugs

The first batch of policy-related bid-winning drugs (*n* = 25) and alternative drugs (*n* = 56) that have an alternative relationship with bid-winning drugs in clinical use were included as study subjects. According to the Reference Monitoring Range of Alternative Drugs of NVBP latest issued by NHSA, alternative drugs are considered to be therapeutic equivalents with different active pharmaceutical ingredients but the same administration route and alternative varieties were sorted into three categories: alternatives drug products with perfect clinical equivalence, alternatives drug products with fundamental clinical equivalence, and alternatives drug products with limited clinical equivalence [15]. For example, the alternative drugs of Escitalopram Oxalate were shown in Table 1. Among them, several drugs were procured in subsequent batches of NVBP during our observation period or for which no procurement records were queried and therefore were excluded from our sample. In total, 56 alternative drugs were included in the analysis. Besides, promoting the replacement of branded drugs with generics is the core of policy design. Thus, non-winning drugs with the same generic name were also dichotomized into branded and generic products in this study (“generic products” in this study refer to “branded generics”) (Fig. 1). Meanwhile, bid-winning drugs were aggregated into 8 ATC groups: A-alimentary tract and metabolism (*n* = 1), B-blood and blood forming organs(*n* = 1), C-cardiovascular system (*n* = 8), N-nervous system (*n* = 7), J-anti-infectives for systemic use(*n* = 3), L-antineoplastic and immunomodulating agents (*n* = 3), M-musculoskeletal system (*n* = 1), and R-respiratory system (*n* = 1). Supplementary Appendix Table A1 lists the policy-related drugs.

**Table 1:** The classification of Escitalopram Oxalate

Bid-winning drug products	Alternatives drug products with perfect clinical equivalence	Alternatives drug products with fundamental clinical equivalence	Alternatives drug products with limited clinical equivalence
Escitalopram Oxalate (Hunan DongtingPharm.Co.Ltd)	Citalopram and Escitalopram Oxalate (Other pharmaceutical manufacturers)	Doxepin, Clomipramine	Fluvoxoxamine, Bupropion, Trazodone, Milnacipran, Mirtazapine, Agomelatine, Amitriptyline

### Outcome measures

We used three outcome indicators in this study: volume, expenditures, and daily cost. To standardize drug use, we applied the defined daily dose (DDD) as the unit of measurement to ensure comparable use of different drugs, according to the Guidelines for ATC classification and DDD assignment 2022 [16]. The DDD value of some medications, which could not be coded in WHO’s system, was determined based on the New Pharmacology (18th Edition) or the recommended adult dosage in the manufacturers’ instructions [17]. Volume was measured using Defined Daily Doses (DDDs), which was calculated by dividing the sales data in volume by the DDD. Expenditure data was reported in CNY. The daily cost was assessed by Defined Daily Drug cost (DDDc), which was estimated as the Expenditures/DDDs.

### Statistical analysis

The NVBP is gradually becoming normalized, and the evaluation of the policy should focus on both its instantaneous and long-term effects. By analyzing the utilization of policy-related drugs, the sustainability of the policy can be evaluated. To analyze trends in the volume, expenditures, and price of policy-related drugs, we performed a single-group Interrupted Time Series (ITS) from December 2018 through December 2020. As the NVBP was implemented on 1 January 2020, in this study, we determined December 2019 as the policy intervention point of the bid-winning drugs and January 2020 as the implementation time point of the NVBP, considering the need for hospitals to purchase drugs in advance. To estimate changes in the levels and trends of each outcome variable after NVBP, the ITS model formula can be expressed as follows:

$${\mathrm{Y}}_{\mathrm{t}}={\upbeta }_{0}+{\upbeta }_{1}*{\mathrm{time}}_{\mathrm{t}} +{\upbeta }_{2}*\mathrm{level}+{\upbeta }_{3}*\mathrm{ trend }+{\mathrm{e}}_{\mathrm{t}}$$

where Y_t_ refers to outcome variables (volume, expenditures, or DDDc) in month t; time_t_ is a continuous variable representing time trend; level represents a dummy variable for the policy intervention; the trend is an interaction term between time and level, and the e_t_ is an estimate of the random error term. In this model, β_0_ estimates the baseline level at time = 0, β_1_, β_2_, β_3_, represents the trend prior to intervention, the level change that occurs immediately after the intervention, and the slope changes caused by the intervention, respectively. The Durbin-Watson test was examined to test the presence of first-order auto-correlation, and *p* < 0.05 was considered statistically significant.

## Results

### The overall situation of policy-related drugs

#### Volume

**Fig. 2:**
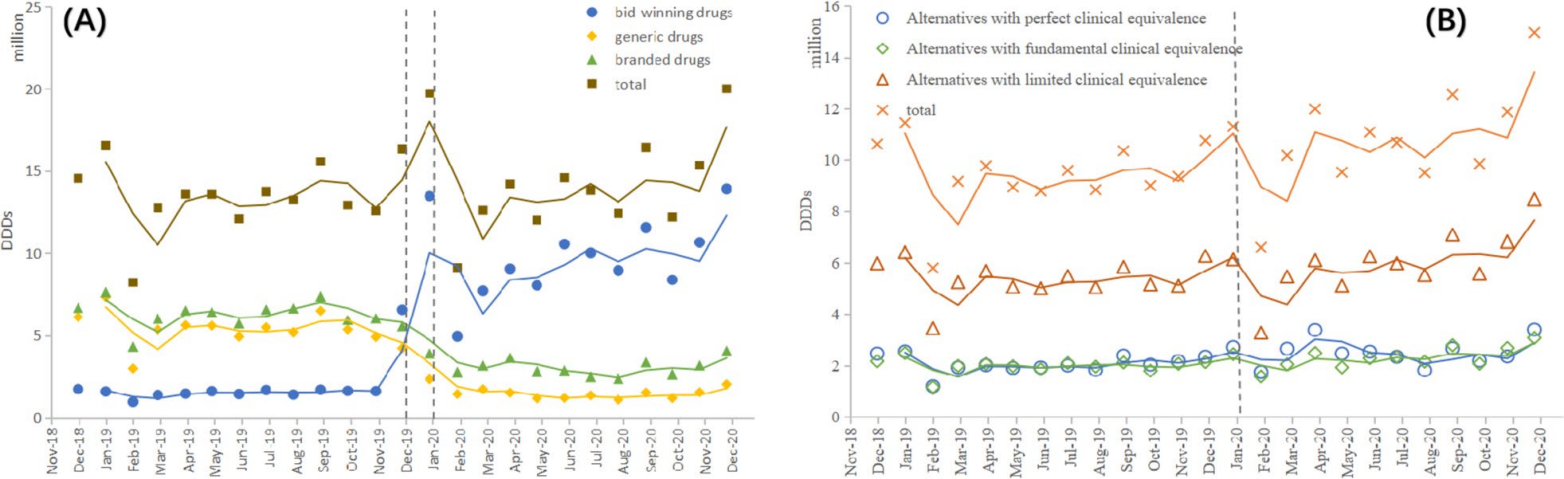
Monthly trends in the volume of bid-winning and alternative products. **A** The trend in the volume of bid-winning drugs and non-winning drugs under the same generic name, **B** The trend in the volume of alternative drugs at different degrees (under different generic names)

The monthly trends in the volume of bid-winning and alternative drugs are displayed in Fig. 2 and Table 2 presents the results of ITS analysis for procurement volume. After the implementation of NVBP, a significant increase was associated with the volume of bid-winning drugs (*p* < 0.001) and the trend change was statistically significant (*p* = 0.037), with an upward trend. The volume of generic and branded drugs decreased immediately after policy intervention (*p* < 0.001), but there was no sustained downward trend following. In aggregated analysis, the volume of drugs under the same generic name remained stable.

**Table 2:** The results of interrupted time series analysis for volume (thousand DDD)

Categories	Constant β_0_(Yt_0_)	Secular trend β_1_(Yt_1_)	Level change β_2_(Yt_2_)	Trend change β_3_(Yt_3_)
**Drugs under the same generic name**				
Bid-winning drugs	1415.77^*^ (1728.26)	9.38 (1502.43)	6344.53^***^ (6560.82)	256.05^*^ (9516.16)
Generic drugs	5657.35^***^ (6112.04)	-49.73 (5345.35)	-3455.25^***^ (2341.37)	32.70 (1496.59)
Branded drugs	6347.83^***^ (6691.72)	-12.07 (6264.50)	-3103.20^***^ (3899.19)	8.71 (3098.93)
Total	13200.00^***^ (14532.02)	22.47 (13266.23)	325.97 (19711.14)	86.26 (14357.97)
**Alternative drugs under different generic names**				
Perfect clinical equivalence	1995.31^***^(2467.62)	10.39 (2057.63)	317.67 (2718.95)	3.76 (2525.83)
Fundamental clinical equivalence	1941.59^***^ (2170.93)	8.37 (1990.46)	-109.23 (2444.98)	58.4^*^ (2325.32)
Limited clinical equivalence	5205.06^***^ (5983.09)	27.00 (5373.02)	-792.40 (6142.75)	189.23^*^ (5990.98)
Total	9114.10^***^ (10621.64)	49.01 (9421.11)	-569.64 (11306.68)	240.48 (10842.14)

^***^p-value < 0.001

^**^p-value < 0.01

^*^p-value < 0.05

Yt_0_ refers to the Yt value of volume in the first month included in the study

Yt_1_ refers to the average volume before policy intervention

Yt_2_ refers to the value of volume in the month of policy intervention

Yt_3_ refers to the average volume after policy intervention

Among the alternative drugs, the procurement volume of neither alternatives with perfect clinical equivalence (*p* = 0.312), alternatives with fundamental clinical equivalence (*p* = 0.564) nor alternatives with limited clinical equivalence (*p* = 0.156) have not been affected by the policy in the instant. However, the long-term trend following the policy intervention showed an upward trend for both alternatives with fundamental clinical equivalence (*p* = 0.029) and limited clinical equivalence (*p* = 0.016). As for the volume of overall alternative drugs, no significant changes were observed for the level or trend.

### Defined daily drug cost

**Fig. 3:**
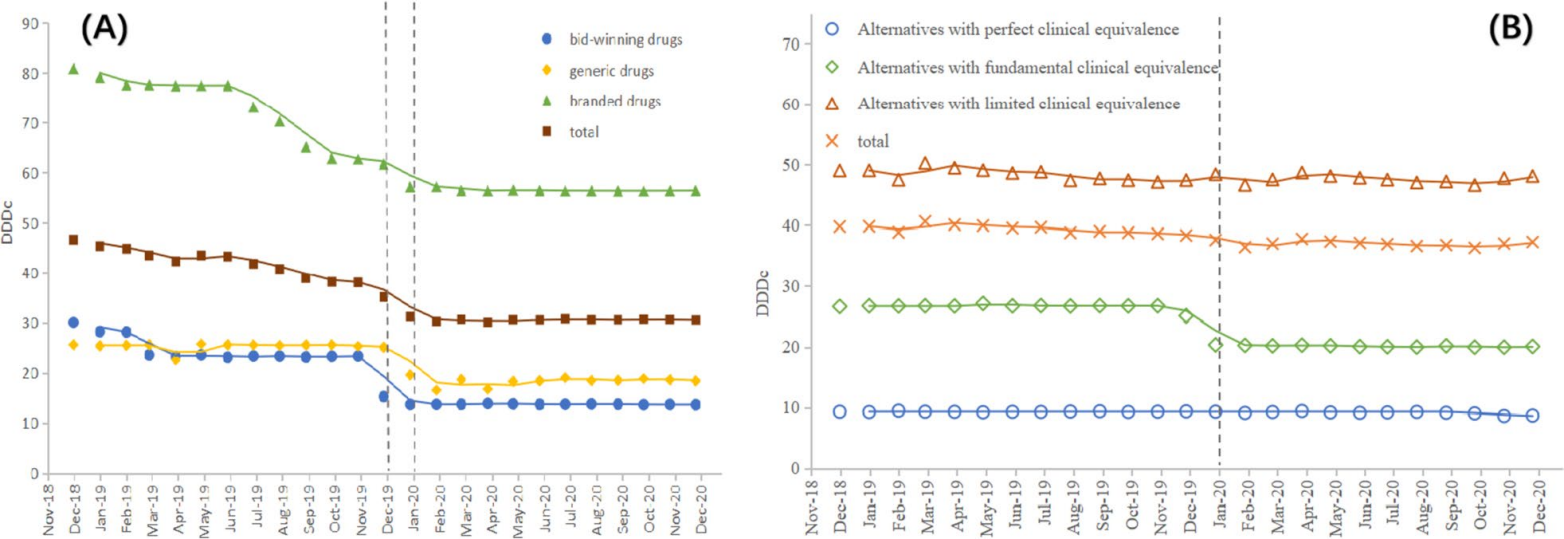
Monthly trends in the DDDc of bid-winning and alternative products. **A** The trend in the DDDc of bid-winning drugs and non-winning drugs under the same generic name, **B** The trend in the DDDc of alternative drugs at different degrees (under different generic names)

Figure 3 outlines the monthly price change of bid-winning drugs and their alternative drugs. The corresponding ITS results regarding the change of DDDc are summarized in Table 3. The trend of bid-winning drugs is continuously down and has a rapid decline (down by 25%, *p* < 0.001) at the time of the policy intervention, and the trend narrowed after the policy. After the policy implementation, the DDDc of generic and branded products showed a sudden decrease, with generic drugs dropping by 7.62 CNY (*p* < 0.001) and branded drugs by 3.07 CNY (*p* = 0.034). Besides, there was a downward trend in branded drugs before the NVBP, and this trend continued after the policy was implemented. As for overall drugs under the same generic name, the DDDc dropped significantly affected by the policy, which was basically consistent with the trend of bid-winning drugs.

**Table 3:** The results of interrupted time series analysis for DDDc

Categories	Constant β_0_(Yt_0_)	Secular trend β_1_(Yt_1_)	Level change β_2_(Yt_2_)	Trend change β_3_(Yt_3_)
**Drugs under the same generic name**				
Bid-winning drugs	28.20^***^ (29.99)	-0.59^*^ (24.64)	-7.12^***^ (15.25)	0.56^*^ (13.84)
Generic drugs	25.04^***^ (25.54)	0.03 (25.21)	-7.62^***^ (19.55)	0.05 (18.32)
Branded drugs	82.04^***^ (80.92)	-1.65^***^ (72.60)	-3.08^*^ (57.21)	1.54^***^ (56.57)
Total	46.72^***^ (46.61)	-0.89^***^ (41.69)	-3.01^**^(31.17)	0.60^**^ (30.53)
**Alternative drugs under different generic names**				
Perfect clinical equivalence	9.31^***^ (9.32)	0.00 (9.33)	0.03 (9.33)	-0.05^*^ (9.13)
Fundamental clinical equivalence	26.91^***^ (26.61)	-0.05^*^ (26.60)	-6.02^***^ (20.34)	0.02 (20.12)
Limited clinical equivalence	49.41^***^ (49.02)	-0.17^**^ (48.37)	0.65 (48.39)	0.14 (47.61)
Total	40.12^***^ (39.78)	-0.13^**^ (39.36)	-1.28^**^ (37.59)	0.09 (37.01)

^***^p-value < 0.001

^**^p-value < 0.01

^*^p-value < 0.05

Yt_0_ refers to the Yt value of DDDc in the first month included in the study

Yt_1_ refers to the average DDDc before policy intervention

Yt_2_ refers to the value of DDDc in the month of policy intervention

Yt_3_ refers to the average DDDc after policy intervention

Among the other alternative drugs, the DDDc change of alternatives with fundamental clinical equivalence was larger, falling by 6.02 CNY (*p* < 0.001) due to the policy. Before the intervention, the DDDc of alternatives with fundamental clinical equivalence (*p* = 0.046) and limited clinical equivalence (*p* = 0.004) both had a slight decline and alternatives with perfect clinical equivalence decreased gently in the post-policy.

### Expenditures

**Fig. 4:**
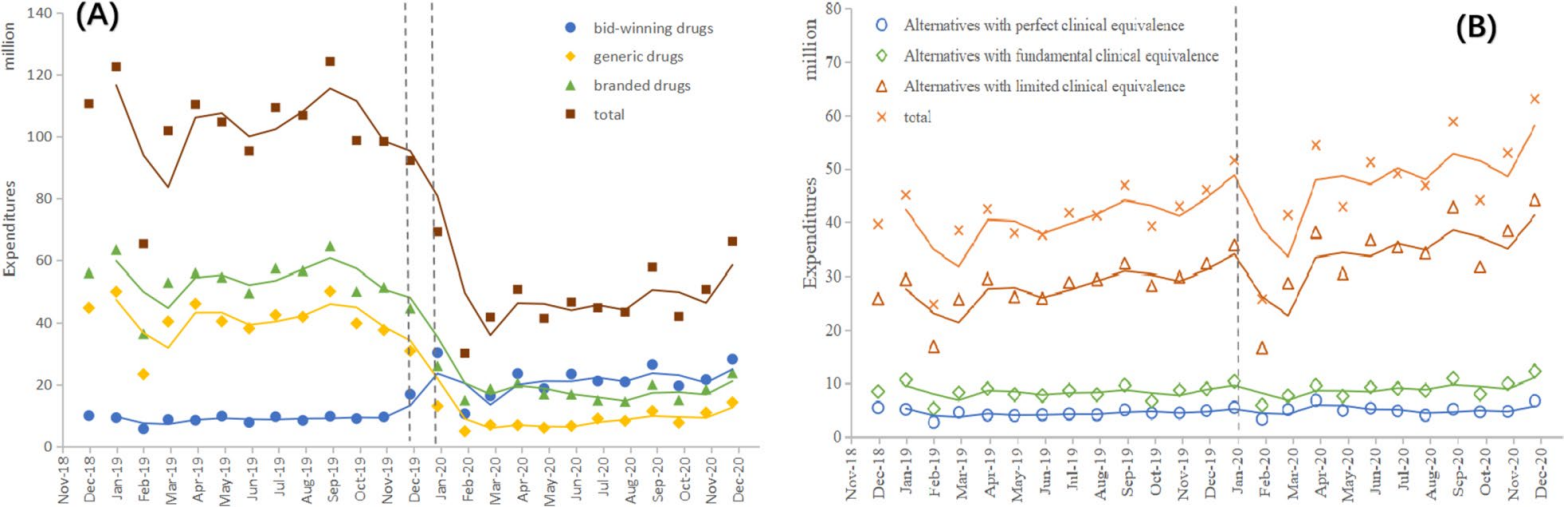
Monthly trends in the expenditures of bid-winning and alternative products. **A** The trend in the expenditures of bid-winning drugs and non-winning drugs under the same generic name, **B** The trend in the expenditures of alternative drugs at different degrees (under different generic names)

Figure 4 shows that with the increase in the volume of the bid-winning drugs, procurement expenditures increased substantially at the time of NVBP implementation. Meanwhile, there were abrupt declines in the expenditures of non-winning generic and branded drugs after policy intervention. Branded drugs dropped by 35.5 million CNY (*p* < 0.001), while generic products decreased by 33 million CNY (*p* < 0.001) (Table 4). Of note, the total expenditures of drugs under the same generic name significantly decreased by 48.2 million CNY after the policy. For alternative varieties, the change in the pre-and post-NVBP slopes and levels had no significant difference, except for a small increase in the alternatives with limited clinical equivalence before the policy.

**Table 4:** The results of interrupted time series analysis for expenditures (million CNY)

Categories	Constant β_0_(Yt_0_)	Secular trend β_1_(Yt_1_)	Level change β_2_(Yt_2_)	Trend change β_3_(Yt_3_)
**Drugs under the same generic name**				
Bid-winning drugs	8.21^***^ (9.84)	0.08 (8.71)	9.57^***^ (16.76)	0.34 (21.25)
Generic drugs	41.80^***^ (44.71)	-0.21 (40.37)	-33.00^***^ (12.90)	0.65 (8.70)
Branded drugs	54.40^***^ (55.95)	-0.09 (53.30)	-35.50^***^ (25.96)	0.03 (18.31)
Total	102.00^***^ (110.49)	0.27 (103.00)	-48.20^**^ (69.05)	-1.17 (48.63)
**Alternative drugs under different generic names**				
Perfect clinical equivalence	4.28^***^ (5.40)	0.01 (4.35)	0.37 (5.44)	0.03 (5.04)
Fundamental clinical equivalence	8.12^***^ (8.42)	0.03 (8.25)	-0.81 (10.33)	0.21 (9.05)
Limited clinical equivalence	23.70^***^ (25.76)	0.67^*^ (27.69)	-5.05 (35.72)	0.56 (34.39)
Total	36.00^***^ (39.59)	0.73 (40.29)	-5.52 (51.50)	0.77 (48.48)

^***^p-value < 0.001

^**^p-value < 0.01

^*^p-value < 0.05

Yt_0_ refers to the Yt value of expenditures in the first month included in the study

Yt_1_ refers to the average expenditures before policy intervention

Yt_2_ refers to the value of expenditures in the month of policy intervention

Yt_3_ refers to the average expenditures after policy intervention

### Changes in policy-related drugs stratified by therapeutic categories

#### Volume

Table 5 summarizes the key results, focusing on the level and trend change of each of the subgroups. After policy intervention, the volume of bid-winning drugs significantly increased in all categories except the nervous system. The volume of generic and branded drugs in most therapeutic categories decreased significantly under the policy intervention (*p* < 0.001), but branded drugs for the antineoplastic and immunomodulating agents showed no statistical decrease. The overall volume change of drugs under the same generic name had no significance (all *p*-values > 0.05).

**Table 5:** Subgroup analyses on the impacts of volume (thousand DDD)

Categories	Drugs under the same generic name				Alternative drugs			
	Bid-winning	Generic	Branded	Total	Perfect	Fundamental	Limited	Total
**ATC-C**								
Level change	4671.36^***^	-2300.95^***^	-2258.69^***^	295.69	329.47	-22.32	-0.19	0.13
Trend change	201.28^*^	4.41	25.57	73.31	-2.95	46.58^*^	0.11^*^	0.15
**ATC-N**								
Level change	85.73	-115.02^***^	-125.61^***^	-86.42	-13.79^*^	-13.67	-0.53^*^	-557.76^*^
Trend change	8.86	6.74^*^	-0.67	9.07	0.86	-0.21	0.05	47.70
**ATC-J**								
Level change	829.67^***^	-497.20^**^^*^	-54.66^***^	246.62	1.30	-70.41	-47.83	-124.64
Trend change	9.76	14.68	-6.70^***^	-3.45	1.04	11.69^*^	31.71^**^	44.60^**^
**ATC-L**								
Level change	38.44^***^	-44.74***	-2.21	-7.15	-	0.43	-0.33	0.06
Trend change	1.29^*^	0.71	0.33	0.65	-	0.61^***^	-0.44	0.17
**Others**								
Level change	722.57^***^	-492.14^***^	-519.28^***^	-171.24	-	-	-8.47	-8.47
Trend change	36.36^**^	5.68	2.86	12.50	-	-	1.60	1.60

^***^p-value < 0.001

^**^p-value < 0.01

^*^p-value < 0.05

“Others” included ATC-A, ATC-B, ATC-M and ATC-R, due to the number of drugs in these categories extremely low

For the alternative drugs for the treatment of nervous diseases, the immediate volume decline was found at the start of the procurement period (*p* = 0.020), with a significant decrease for alternatives drug products with perfect clinical equivalence (Citalopram, *p* = 0.023) and for alternatives drug products with limited clinical equivalence (*p* = 0.021), but no downward trend in the later. In the post-intervention period, the volume of alternatives drug products with fundamental clinical equivalence and limited clinical equivalence in the cardiovascular and anti-infection system showed a “carry-over phenomenon”, while the volume of alternatives with fundamental clinical equivalence in antineoplastic and immunomodulating agents increased (*p* < 0.001).

### Defined daily drug cost

The corresponding ITS results are presented in Table 6. Among 8 therapeutic categories of bid-winning drugs, except for ATC-L (*p* = 0.701), the immediate decline was detected in the DDDc of the other categories at the start of the NVBP (all *p*-values < 0.001). The following trend changes slightly decreased in the DDDc of ATC-C (*p* = 0.013) and ATC-N (*p* = 0.003) during the implementation of the procurement period. Changes in DDDc for most therapeutic categories of non-winning drugs under the same generic name were consistent with the policy influence, while, the branded drugs in the antineoplastic system were somewhat different. There was a downward trend in the DDDc of ATC-L before the policy (*p* < 0.001) and the immediate change had no significance (*p* = 0.573), then the decrease slowed down (β_1_ = -11.45, β_3_ = 11.16).

**Table 6:** Subgroup analyses on the impacts of DDDc

Categories	Drugs under the same generic name				Alternative drugs			
	Bid-winning	Generic	Branded	Total	Perfect	Fundamental	Limited	Total
**ATC-C**								
Level change	-1.89^***^	-1.02^***^	-1.23^***^	-1.18^***^	-0.05^**^	-0.16^***^	-0.01	-0.11^***^
Trend change	-0.07^*^	0.031^*^	-0.00	-0.02	0.01^**^	-0.00	-0.01	-0.00
**ATC-N**								
Level change	-11.53^***^	-4.83^***^	-3.35^***^	-3.83^***^	0.12	0.026	-0.07	0.30^*^
Trend change	-0.36^**^	-0.22	-0.07^**^	-0.28	-0.22^**^	-0.00	0.12	0.01
**ATC-J**								
Level change	-14.14^***^	-3.97^***^	-3.75^***^	-4.23^***^	-0.10	-0.22^*^	-1.49	-0.68
Trend change	-0.04	-0.06	-0.00	-0.16^*^	-0.01	-0.01	-0.11	-0.05
**ATC-L**								
Level change	-4.02	-46.79^***^	-5.36	-10.51	-	-47.14^***^	3.42^*^	-13.29^***^
Trend change	6.06^*^	0.74	11.16^***^	6.23^***^	-	0.22	0.39	0.33
**Others**								
Level change	-14.96^***^	-3.13^***^	-3.67^***^	-5.27^***^	-	-	6.94	6.94
Trend change	0.12^*^	0.11^***^	-0.19	-0.15	-	-	0.84	0.84

^***^p-value < 0.001

^**^p-value < 0.01

^*^p-value < 0.05

“Others” included ATC-A, ATC-B, ATC-M and ATC-R, due to the number of drugs in these categories extremely low

As for alternative drugs, immediate DDDc decreases were observed in the cardiovascular system (β_2_ = -0.11, *p* < 0.001), with the estimated decrement of 0.05 CNY in the alternatives drug products with perfect clinical equivalence (Levamlodipine) and 0.16 CNY in the alternatives with fundamental clinical equivalence at the implementation of NVBP. In terms of the overall alternative drugs in the nervous system, we did not observe significant decrements of DDDc, and even a slight increase was displayed at the intervention of policy. There were abrupt declines in the DDDc of alternatives with fundamental clinical equivalence in the ATC-L (β_2_ = -47.14, *p* < 0.001) as well, but the increment of alternatives with limited clinical equivalence (β_2_ = 3.42, *p* = 0.028) was found. In another drug category, the monthly changes of alternative drugs in DDDc showed no statistical difference affected by the policy.

### Expenditures (thousand CNY)

Table 7 demonstrates the results of drug expenditures among different therapeutic categories. The bid-winning drugs in three (ATC-L, ATC-B, and ATC-R) of the eight therapeutic categories showed significant increments in expenditure level change during the procurement period (all *p*-value < 0.001), while drugs of anti-infectives for systemic use significantly decreased by 364 thousand CNY (*p* = 0.004). A significant decrease in non-winning drugs under the same generic name (including generic and branded drugs) in all therapeutic categories in expenditure level change was detected after the policy intervention. In regards to products under the same generic name, the overall expenditure of bid-winning and non-winning drugs is reduced, except for drugs in the nervous system. Among alternative drugs, notable upward trends were observed for the expenditure of alternatives drug products with fundamental clinical equivalence in ATC-J (β1 = 7.22, β3 = 85.81) and ATC-L (β1 = -50.36, β3 = 91.62) and alternatives with limited clinical equivalence in ATC-C (β1 = 3.33, β3 = 252.08) and ATC-J (β1 = 73.87, β3 = 185.61). When the NVBP policy was implemented, the expenditure of two categories suddenly decreased by 2.61 million CNY (alternatives with limited clinical equivalence in ATC-N) and 0.57 million CNY (alternatives with fundamental clinical equivalence in ATC-J), respectively. The change of other categories in expenditure slope showed no significance.

**Table 7:** Subgroup analyses on the impacts of expenditures (thousand CNY)

Categories	Drugs under the same generic name				Alternative drugs			
	Bid-winning	Generic	Branded	Total	Perfect	Fundamental	Limited	Total
**ATC-C**								
Level change	534.53	-7054.82^***^	-16,600.00^***^	-18,900.00^**^	425.88	-130.16	-565.91	-252.83
Trend change	1.89	167.14	97.71	-513.01	19.66	38.08	252.08^*^	289.16
**ATC-N**								
Level change	1666.28	-5302.76^***^	-1251.70^***^	-3423.59	-87.38	-6.54	-2606.62^**^	-2699.69^**^
Trend change	41.47	207.32^**^	-34.97	16.70	-2.87	-0.29	229.84	227.88
**ATC-J**								
Level change	-364.30^**^	-5386.60^***^	-1170.61^***^	-7234.71^***^	-0.46	-570.38^*^	-426.63	-1070.85
Trend change	24.60	76.77	-81.64^**^	-229.26	10.84	85.81^*^	185.61^**^	282.27^**^
**ATC-L**								
Level change	5610.89^***^	-10,100.00^***^	-830.18^*^	-5107.80^*^	-	-87.39	-236.16	-336.03
Trend change	136.23	96.98	100.62	-51.36	-	91.62^***^	-209.49*	-116.85
**Others**								
Level change	2305.78^***^	-4927.85^***^	-13,900.00^***^	-14,100.00^***^	-	-	-1031.71	-1031.71
Trend change	163.79^**^	94.84	-17.68	-397.73	-	-	99.82	99.82

^***^p-value < 0.001

^**^p-value < 0.01

^*^p-value < 0.05

“Others” included ATC-A, ATC-B, ATC-M and ATC-R, due to the number of drugs in these categories extremely low

## Discussion

This is a study to evaluate the impact of the implementation of the NVBP, quantifying changes in the utilization of the “4 + 7” policy-related drugs in public medical institutions in Nanjing on a monthly basis. The study focused on the direct and indirect effects of the policy, concentrating on the impact on the bid-winning drugs and related alternative drugs, as well as the differences in the drugs of different therapeutic categories. This study provided empirical analysis results for the government to optimize the policy, which helped to improve the effectiveness of policy implementation. It is of great significance for exploring the mechanism of drug price formation under market guidance, and standardizing the drug circulation. Overall, the short-term effects of the policy were obvious, with the volume of the bid-winning drugs increasing and the defined daily drug cost falling. The procurement of various alternative drugs was relatively stable, but the volume of drug utilization occurred spillover effects during the post-policy period. Furthermore, the procurement situation of policy-related drugs in individual therapeutic areas is abnormal.

First of all, the results of this study showed that the volume of bid-winning drugs significantly increased under the impact of the policy implementation, while the opposite was seen in the generic and branded drugs, and the overall procurement volume of drugs under the same generic name remained stable. Also, previous studies have reported similar results [9, 10, 13, 18]. In China, patients were previously more dependent on branded drugs [19] while foreigners had a higher acceptance of generics [20]. For example, generic drugs account for 90 percent of the prescriptions dispensed in the United States [21] and the generic substitution rate increased to 73% in 2018 in Japan [22]. The reason for the low utilization of generic drugs in China may be the fact that patients and physicians are still concerned that generic drugs are clinically inferior to branded drugs. However, the bid-winning drugs are generic drugs that have passed the GCE, which means that the quality and efficacy of generic drugs are consistent with the branded drugs [23]. The NVBP has reshaped the pattern of the drug market with drug use more concentrating on bid-winning drugs and quality-guaranteed drugs and accelerated the substitution of the generic drug. Besides, the study also found that the purchase of individual bid-winning drugs led to a reduction of the volume to zero in some branded drugs (Enalapril Maleate) or generic drugs from other manufacturers (Fosinopril Sodium, Lisinopril, Montelukast Sodium) in the process of policy implementation. This finding may implicate that bid-winning drugs may limit physician prescribing and patient choice of medication to a certain extent. Meanwhile, the shortage of supply from the bid-winning manufacturer will affect the accessibility of the drug to patients. In order to better dispel misgivings on generic drugs and ensure effective implementation of the policy in the future, it is necessary to provide more real-world evidence of clinical consistency of bid-winning generic drugs, and strengthen patients’ understanding of generic drugs, as well as to rationally arrange the purchased quantity of bid-winning drugs to meet the diversity of medication utilization of patients. In the case of emergencies, it is also important to strengthen coordination with purchasers to avoid relying exclusively on a single supplier [24, 25].

Second, we observed significant DDDc reductions of the products under the same generic name and NVBP saved 48.2 million CNY of drug expenditures with the total volume of drugs under the same generic name remaining stable. This reflects the direct price reduction effect of centralized procurement and echoes other research which has reported distinct results on the cost-saving effects of drug policies in different countries [26]. Chaudhury et al.’s study reported that pooled procurement of essential drugs saved the government of Delhi nearly 30% of the annual drugs bill [27]. The research in Mexico mentioned that cost savings through the Price Negotiation for 12 ARV drugs reduced total spending by 38 percent [28]. An international study regarding drug tendering policy by NGOs for cardiovascular and anti-infective drugs showed that the price for tendered originators and generics was 42.4% and 66.8% less than the price for retail respectively [29]. Simultaneously, during the policy implementation period, manufacturers of non-winning drugs consciously lowered prices to gain greater market share under market pressure, with generic drugs falling 7.62 CNY and branded drugs falling 3.07 CNY. With the continuous improvement of the quality of generic drugs in China, there was a downward trend in branded drugs prior to NVBP, and the implementation of the policy resulted in further price reductions. The “patent cliff” for branded drugs has gradually emerged. These findings suggest that centralized purchasing was conducive to reducing health systems costs and improving market competitiveness. In addition, among alternative drugs, the DDDc of alternatives with fundamental clinical equivalence changed greatly, decreasing by 6.02 CNY. This could be explained by a large drop in the DDDc of Erlotinib. In 2020, Roche, the pharmaceutical manufacturer of originator products, reduced the price after the national drug price negotiation [30], and then the first domestic generic manufacturer of Erlotinib (approved in September 2019), Shanghai Acebright Pharma, also entered the market with a lower price. On the other hand, the overall situation for alternative drugs was relatively stable with no upward trend in prices and expenditures as feared, indicating that monitoring of alternative drugs was necessary.

Third, policy effectiveness varies in different therapeutic categories. Among the drug list, drugs for the treatment of cardiovascular diseases accounted for the largest share. Since the volume of drugs under the same generic name is basically unchanged, the reduction in price resulted in a substantial decrease in the total expenditures of ATC-C, indicating the potential policy effect of relieving the medication burden of patients with cardiovascular diseases. The NVBP exerted little influence on drugs for the treatment of nervous diseases, with the price of most bid-winning products did not drop much, no significant increase in the volume of bid-winning drugs, and no decrease in total spending for drugs under the same generic name, as well as the volume of alternative drugs even declined. On the one hand, it might be ascribed to the fewer eligible drugs being negotiated. On the other hand, patients with mental disorders are more dependent on medicines in use and are less willing to switch medications, which may entail risks of substantial side effects [31]. At the same time, the number of patients with mental health problems attending the clinic has decreased due to the outbreak of the COVID-19 epidemic. Nevertheless, different from other classes of drugs, the policy has not achieved the anticipation in the purchase volume of bid-winning drugs in place of originators in the system of antineoplastic and immunomodulating agents, and the volume of non-winning branded drugs did not decline. This may be related to patient loyalty and efficacy differences with generic drugs [32]. In order to further improve the utilization rate of generic drugs in ATC-L, we could increase the confidence and knowledge of patients through medical staff and rigorously assure the quality of generic drugs [33]. Certainly, patients are more concerned about the severity of side effects than physicians, we call for physicians also take patient preferences into account when altering therapeutic decisions [34]. Prior to centralized procurement, a series of policies continued to reduce the price of anticancer drugs, which made the reduction of DDDc of some drugs in ATC-L not statistically significantly affected by NVBP. The government imposed a zero tariff on imported drugs from abroad and price negotiation was also adopted to reduce anticancer drug prices, especially the price of original drugs continued to fall [35].

Lastly, the long-term trend after the policy intervention showed that the volume of alternatives drug products with fundamental clinical equivalence and limited clinical equivalence was on the rise. The procurement volume of alternative drugs appeared to be a “carry-over” mainly in three categories (ATC-C, ATC-J, and ATC-L), the main reason may be that the price of the bid-winning drugs in these three categories fell significantly. The increase in alternative drug volume can be attributed to the “spillover effect”. We observed this phenomenon may potentially exist in some medical institutions, where hospitals received rebates to increase the prescription of more expensive alternative drugs, as the NVBP led to a reduction in pharmaceutical industry profit. Similar results were obtained in the study that there was excessive procurement of related drugs in the early stage of policy implementation [18, 36]. Previous studies have shown how generic substitution may affect different patients differently. With less information available, older patients and those with lower education levels have shown negative attitudes towards generic substitution, resulting in under-dosing and overuse, and reduced adherence. At the same time, pharmacists have expressed concern about policies of generic substitution that make it harder for pharmacists to track patients' medications [37]. Therefore, it is necessary to help patients obtain the necessary knowledge, reduce their concerns, and take measures to strengthen the monitoring of patients using of generic drugs to ensure the implementation of the generic drug substitution policy [38]. This also prompted us to pay attention to the rationality of the procurement behavior of some medical institutions after the centralized procurement policy, which was fundamental for minimizing spillover effects from alternative drug interventions across hospitals. Moreover, we should not reduce drug prices blindly and it is necessary to maintain reasonable profits for pharmaceutical factories to promote the development of the domestic pharmaceutical industries and pharmaceutical innovation.

There are several potential limitations regarding the study. First, this study is confined to the city of Nanjing, which restricts the generalizability of the findings to a broader context. The findings may not be fully representative of the overall implementation of the NVBP in China. Considering the differences in economic levels and medication habits in different regions, caution should be exercised in extrapolating the findings. Therefore, to increase the sample representativeness, more cities and areas should be included in the future study. Second, although the use of ITS analysis allows for the consideration of time-related factors, it is possible that potential confounding variables have not been fully accounted for. In subsequent studies, using a multiple-group ITS for in-depth analysis can effectively identify the net effects of interventions, as this method involves a control group. Third, due to the accessibility of data, the dataset only spans part of the alternative drugs of the monitoring program, resulting in certain defects in this study. In future research, we will consider consulting clinical doctors and pharmacists to include more alternative drugs based on their indications, clinical frequency of use, and other characteristics to improve this study. Besides, this study was based on drug purchase data, which may not precisely match the drug use data, although the two have been shown to be strongly consistent. In spite of these limitations, this study systematically analyzed the effect of NVBP on the use of policy-related drugs in the area outside the pilot phase by the latest monitoring list and can better reflect the situation in Nanjing hospitals and thus did reveal the current situation of the policy. It is necessary to further discover the long-term impact of NVBP on the medical industry through research on drug utilization, and evaluate whether the decline in drug prices is sustainable.

## Conclusion

This study assessed the impact of the NVBP on the use of policy-related drugs, providing evidence that the policy effectively lowered the price, increased the volume of the bid-winning drugs, and promoted generic substitution. Meanwhile, the policy drove down the price of non-winning drugs under the same generic name, saving the total cost of the policy-related drugs. The policy effectiveness varies in different therapeutic categories. For alternative drugs, there was a “spillover effect” in the volume after the policy was implemented. To improve the policy, policy-related drugs deserve full attention in policy monitoring and reasonable profits should be maintained for pharmaceutical factories. On the other hand, it is necessary to raise patients’ awareness of generic drugs and ensure the quality of generics.

### Supplementary Information

**Additional file 1:** **Appendix Table 1. **Description of the bid-winning drugs and alternative drugs. 

## Data Availability

The original data in this study are included in the article and further inquiries can be directed to the corresponding authors.

## Abbreviations

NHSA: National Healthcare Security Administration
NVBP: National volume-based procurement policy
GCE: Generic Consistency Evaluation
DDD: Defined daily dose
DDDs: Defined Daily Doses
DDDc: Defined Daily Drug cost
ITS: Interrupted Time Series
