# Supplementary material for: The impact of the national volume-based procurement policy on the use of policy-related drugs in Nanjing: an interrupted time-series analysis
Source: Int J Equity Health. 2023 Sep 28;22:200. doi: 10.1186/s12939-023-02006-1 (PMC10540346; doi:10.1186/s12939-023-02006-1)
Supplement: Supplementary file 1 — Additional file 1: Appendix Table 1. Description of the bid-winning drugs and alternative drugs. [file 12939_2023_2006_MOESM1_ESM.docx]

**Appendix Table 1. Description of the bid-winning drugs and alternative drugs**

|  | **Generic Names** | **DDD** |
| --- | --- | --- |
| **“4+7” policy-related varieties** | Atorvastatin Calcium | 20 |
|  | Rosuvastatin Calcium | 10 |
|  | Clopidogrel Bisulfate | 75 |
|  | Irbesartan | 150 |
|  | Amlodipine Besylate | 5 |
|  | Entecavir | 0.5 |
|  | Escitalopram Oxalate | 10 |
|  | Paroxetine Hydrochloride | 20 |
|  | Olanzapine | 10 |
|  | Cefuroxime Axetil | 500 |
|  | Risperidone | 6 |
|  | Gefitinib | 250 |
|  | Fosinopril Sodium | 10 |
|  | Irbesartan and Hydrochlorothiazide | 162.5 |
|  | Lisinopril | 10 |
|  | Tenofovir Disoproxil Fumarate | 300 |
|  | Losartan Potassium | 50 |
|  | Enalapril Maleate | 10 |
|  | Levetiracetam | 1000 |
|  | Imatinib Mesylate | 400 |
|  | Montelukast Sodium | 10 |
|  | Montmorillonite Powder | 9000 |
|  | Pemetrexed Disodium | 24 |
|  | Flurbiprofen Axetil | 50 |
|  | Dexmedetomidine Hydrochloride | 0.2 |
| **Alternatives drug products with perfect clinical equivalence** | Levamlodipine | 2.50 |
|  | Lamivudine | 100 |
|  | Telbivudine | 600 |
|  | Citalopram | 20 |
| **Alternatives drug products with fundamental clinical equivalence** | Pravastatin | 40 |
|  | Fluvastatin | 20 |
|  | Telmisartan | 40 |
|  | Allisartan Isoproxil | 240 |
|  | Doxepin | 50 |
|  | Clomipramine | 50 |
|  | Penfluridol | 20/seven days |
|  | Cefprozil | 500 |
|  | Erlotinib | 150 |
|  | Icotinib | 375 |
|  | Ramipril | 2.5 |
|  | Losartan Potassium and Hydrochlorothiazide | 62.5 |
|  | Telmisartan and Hydrochlorothiazide | 92.5 |
|  | Cilexetil and Hydrochlorothiazide | 28.5 |
|  | Olmesartan Medoxomil and Hydrochlorothiazide | 32.5 |
|  | Benazepril | 10 |
| **Alternatives drug products with limited clinical equivalence** | Felodipine | 5 |
|  | Nifedipine | 30 |
|  | Lacidipine | 2 |
|  | Nitrendipine | 10 |
|  | Benidipine | 2 |
|  | Fluvoxoxamine | 100 |
|  | Bupropion | 150 |
|  | Trazodone | 150 |
|  | Duloxetine | 40 |
|  | Milnacipran | 50 |
|  | Mirtazapine | 15 |
|  | Agomelatine | 25 |
|  | Amitriptyline | 50 |
|  | Amisulpride | 400 |
|  | Haloperidol | 10 |
|  | Aripiprazole | 10 |
|  | Paliperidone | 6 |
|  | Ziprasidone | 40 |
|  | Chlorpromazine | 50 |
|  | Cefixime | 100 |
|  | Cefadroxil | 1000 |
|  | Sulpiride | 200 |
|  | Afatinib | 40 |
|  | Osimertinib | 80 |
|  | Oxcarbazepine | 600 |
|  | Magnesium Valproate | 400 |
|  | Topiramate | 100 |
|  | Sodium Valproate | 600 |
|  | Carbamazepine | 100 |
|  | Lamotrigine | 50 |
|  | Nilotinib | 800 |
|  | Dasatinib | 100 |
|  | Parecoxib | 60 |
|  | Diclofenac | 75 |
|  | Ketorolac tromethamine | 120 |
|  | Midazolam | 5 |

DDD, defined daily dose.
